# Apolipoprotein B/A1 Ratio Improves Discrimination of Severe Atherosclerosis Beyond Conventional Lipid Markers in High-Risk Statin-Naive Patients

**DOI:** 10.3390/jcm15145598

**Published:** 2026-07-16

**Authors:** Taedong Ok, Woo-Ri Lee, Sung Hun Kim, Kwon-Duk Seo

**Affiliations:** 1 Department of Neurology, National Health Insurance Service Ilsan Hospital, Goyang 10444, Republic of Korea; gentleboyn@gmail.com; 2 Department of Research and Analysis, National Health Insurance Service Ilsan Hospital, Goyang 10444, Republic of Korea; wind0747@gmail.com; 3 Department of Neurology, Kangwon National University Hospital, Kangwon National University School of Medicine, Chuncheon 24289, Republic of Korea; marinen@kangwon.ac.kr; 4 Department of Radiology, Gangnam Severance Hospital, Yonsei University College of Medicine, Seoul 06273, Republic of Korea

**Keywords:** apolipoproteins, atherosclerosis, carotid stenosis, intracranial arteriosclerosis, risk stratification, lipid biomarkers

## Abstract

**Background/Objectives:** The apolipoprotein B to apolipoprotein A1 (ApoB/A1) ratio has been proposed as a marker of cardiovascular risk, but its value for assessing extracranial and intracranial atherosclerosis beyond conventional lipid markers remains uncertain. We evaluated whether the ApoB/A1 ratio improves discrimination of severe atherosclerosis in high-risk statin-naive patients. **Methods:** We retrospectively included 3416 statin-naive patients who underwent lipid profiling and carotid duplex ultrasonography and/or cerebrovascular imaging between 2014 and 2023. The primary outcome was stenosis severity, categorized as normal, mild (<50% stenosis), or severe (≥50% stenosis, including occlusion), based on clinical imaging reports. Secondary outcomes were carotid plaque score and the number of stenotic intracranial vessels. Associations were assessed using multivariable ordinal logistic regression with 1-SD–standardized lipid markers. **Results:** The ApoB/A1 ratio was independently associated with greater stenosis severity (adjusted odds ratio 1.24 per 1-SD increase, 95% CI 1.15–1.35; *p* < 0.0001). Adding ApoB/A1 to a model including clinical covariates, LDL-C, and HDL-C significantly improved discrimination of severe stenosis on every metric examined (continuous NRI +0.142, 95% CI 0.053–0.227; IDI +0.0058, 0.0026–0.0088; likelihood ratio *p* = 0.0004), although the absolute increase in AUC was modest (0.660 to 0.668; ΔAUC +0.008; DeLong *p* = 0.026). Associations were consistent for carotid plaque score and intracranial vessel-count outcomes. **Conclusions:** In high-risk statin-naive patients undergoing carotid or cerebrovascular evaluation, the ApoB/A1 ratio was independently associated with atherosclerosis severity and provided modest incremental discrimination beyond conventional lipid markers. Apolipoprotein assessment may help refine risk stratification in this population.

## 1. Introduction

Dyslipidemia is a major risk factor for atherosclerosis [1]. Dyslipidemia is conventionally defined when total cholesterol, low-density lipoprotein cholesterol (LDL-C), or triglycerides exceed reference values, or when high-density lipoprotein cholesterol (HDL-C) falls below reference values [2]. Among these, LDL-C is the primary target for lipid-lowering therapy to mitigate atherosclerotic cardiovascular disease [3]. However, residual cardiovascular risk persists in many patients despite achieving recommended LDL-C levels, prompting the search for additional biomarkers [4].

LDL-C is composed of subtypes that differ in atherogenicity. Small dense LDL particles, characterized by the highest density and smallest size, contribute critically to atherosclerosis [5]. Apolipoprotein B (ApoB) is exclusively located on the surface of all atherogenic lipoproteins (LDL, very-low-density lipoprotein, and intermediate-density lipoprotein) and plays a central role in lipoprotein metabolism [6]. ApoB enhances the adhesion of LDL particles to arterial endothelial receptors and facilitates their attachment to the arterial wall, ultimately promoting atherosclerotic plaque formation [7]. Conversely, apolipoprotein A1 (ApoA1), a major component of HDL, contributes to cholesterol efflux from peripheral tissues, including the vasculature, thereby reducing atherosclerotic risk [8].

ApoB has been shown to be a superior marker of acute myocardial infarction risk compared with total cholesterol or LDL-C, as it reflects the concentration of all atherogenic lipoproteins [9,10]. Previous studies have demonstrated significant associations between ApoB and the ApoB/A1 ratio and carotid atherosclerosis or unstable plaque [11,12], and a high ApoB/A1 ratio has been linked to intracranial atherosclerotic stenosis [13,14]. However, prior work has typically been limited to single vascular territories, used binary definitions of stenosis, or included substantial proportions of patients on statins, each of which may obscure the relationship between apolipoproteins and atherosclerosis burden. Therefore, the incremental value of the ApoB/A1 ratio in patients undergoing concurrent carotid and cerebrovascular evaluation remains uncertain.

In this study, we evaluated whether the ApoB/A1 ratio provides discriminatory value beyond conventional lipid markers for clinically significant atherosclerosis in high-risk statin-naive patients undergoing carotid or cerebrovascular evaluation.

## 2. Materials and Methods

### 2.1. Data Source and Study Population

This retrospective study used a hospital-based clinical cohort rather than a population-based screening sample. Patients were eligible if they underwent lipid profiling and at least one clinically indicated vascular assessment, including carotid duplex ultrasonography and/or cerebrovascular MRA/CTA, within one year of lipid measurement. Thus, patients classified as having “normal” vessels were those without definite stenosis on the available clinical vascular assessment, not healthy controls or asymptomatic volunteers. The study therefore evaluates discrimination within a high-risk clinical population undergoing routine vascular evaluation and does not address normative ApoB/A1 variation or screening performance in the general population.

Patients were identified from the clinical data warehouse of the National Health Insurance Service Ilsan Hospital, a comprehensive database containing electronic medical records and laboratory results. 

**Figure 1:**
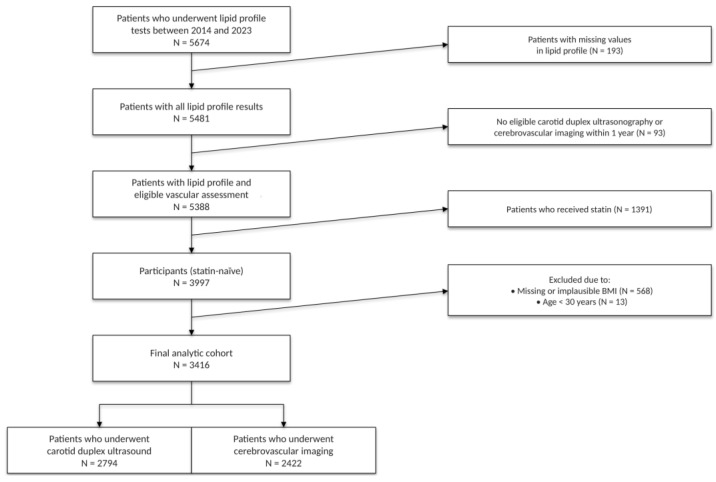
Patient flow diagram showing derivation of the analytic cohort from the source database.

Between January 2014 and December 2023, a total of 5674 patients underwent lipid profile testing. As detailed in the patient flow diagram (Figure 1), after excluding patients with missing lipid profile values (n = 193) and those without an eligible vascular assessment, defined as carotid duplex ultrasonography (CUS) or cerebrovascular imaging with magnetic resonance angiography (MRA) or computed tomography angiography (CTA), within one year of lipid measurement (n = 93), we identified 3997 statin-naive patients who had not received statin therapy for at least three months prior to testing. From these 3997 candidates, we further excluded 568 patients with missing or implausible BMI (outside 15–50 kg/m^2^) and 13 patients younger than 30 years, yielding a final analytic cohort of 3416 patients. Of these, 2794 underwent CUS and 2422 underwent cerebrovascular imaging (Figure 1). 

This study was conducted in accordance with the Declaration of Helsinki (2013 revision) and approved by the Institutional Review Board of the National Health Insurance Service Ilsan Hospital (protocol code NHIMC-2024-04-024, approved 24 April 2024). Written informed consent was waived because de-identified data were used.

### 2.2. Outcomes and Covariates

Atherosclerosis severity was assessed in three complementary ways. The primary outcome was the maximal degree of stenosis, categorized as normal (no stenosis), mild (<50% stenosis), or severe (≥50% stenosis, including occlusion) based on severity descriptors documented in clinical imaging reports. These categories reflect radiologists’ qualitative reporting at the time of original clinical interpretation rather than independently re-measured stenosis percentages. Throughout the manuscript, the term “normal” refers only to the absence of definite stenosis on the index vascular assessment and should not be interpreted as absence of vascular risk factors or clinical disease. Key terms describing vascular segment and severity were extracted from imaging reports using a rule-based text-parsing approach and converted into the quantitative indicators used in analysis. The rule-based extraction process was developed through iterative manual review of imaging reports. During data cleaning, more than 100 reports were manually compared with the extracted vascular segments and severity categories, and the rules were refined to address discordant or ambiguous expressions. The final rule set defined negative expressions, stenosis severity descriptors, eligible intracranial vascular segments, laterality assignment, duplicate-removal rules, and exclusion terms for infarct-territory or extracranial expressions. Details of the extraction rules and representative report expressions with their assigned categories are provided in Supplementary Methods and Supplementary Table S1. Because the manually reviewed reports were used for rule refinement rather than as an independent validation dataset, formal agreement statistics were not calculated.

Two burden-based secondary outcomes were also assessed. Extracranial atherosclerosis burden was quantified using the carotid plaque score (CPS), defined as the sum of plaque thicknesses identified on CUS [15]. Patients with CPS = 0 were classified as normal; the remaining patients were stratified into mild and severe based on the median CPS among those with any plaque (median = 3.0). Intracranial atherosclerosis burden was quantified by the number of vessels with any stenosis (range, 0–11) across both left and right distal internal carotid, anterior cerebral, middle cerebral, posterior cerebral, distal vertebral, and basilar arteries [16]. Patients without stenosis formed the normal group, and the remainder were stratified into mild and severe based on the median vessel count among those with stenosis (median = 3).

The lipid panel comprised total cholesterol, LDL-C, HDL-C, triglycerides, ApoA1, and ApoB. Non-HDL-C and the ApoB/A1 ratio were calculated as derived markers [17,18]. Covariates considered as confounders were age, sex, BMI, history of hypertension, diabetes mellitus, chronic kidney disease, prior stroke, prior ischemic heart disease, alcohol consumption, and current smoking.

### 2.3. Statistical Analysis

Continuous variables are presented as mean ± standard deviation when approximately normally distributed and as median (interquartile range, IQR) otherwise; categorical variables are presented as n (%). Group comparisons used analysis of variance, the Kruskal–Wallis test, or the chi-squared test as appropriate.

Because established thresholds for CPS and the number of stenotic intracranial vessels are lacking, patients with any plaque or stenosis were divided into mild and severe groups using the median value. Tertile-based cutoffs were examined in sensitivity analyses. Associations between each lipid biomarker and atherosclerosis severity were estimated using multivariable ordinal logistic regression, adjusting for the covariates listed above. To enable direct comparison across markers with different scales, all lipid biomarkers were standardized, and effect sizes are reported as adjusted odds ratios per 1-standard-deviation (SD) increase. Because lipid biomarkers exhibited strong collinearity (variance inflation factor > 5 and infinite for derived measures), each biomarker was entered into a separate model rather than simultaneously. The proportional odds assumption was assessed for ordinal logistic regression models.

Incremental discrimination was assessed by comparing a reference model including clinical covariates, LDL-C, and HDL-C with an extended model additionally including the ApoB/A1 ratio. Model performance was compared using AUC with the DeLong test [19], continuous NRI, IDI, and likelihood ratio testing [20]. Bootstrap resampling was used to estimate 95% confidence intervals for NRI and IDI. Alternative reference models incorporating triglycerides or non-HDL-C were evaluated as sensitivity analyses. Restricted cubic splines with four knots were used to assess potential non-linearity between the ApoB/A1 ratio and each outcome.

Subgroup analyses were performed by sex, age (<65 vs. ≥65 years), hypertension, diabetes, and smoking. Effect heterogeneity was assessed by including a multiplicative interaction term in the ordinal model. Sensitivity analyses examined (i) patients who underwent both CUS and cerebrovascular imaging and (ii) tertile-based rather than median-based severity cut-offs. Two-sided *p* < 0.05 was considered statistically significant. Analyses were performed in Python 3.10 using statsmodels, scikit-learn, and scipy.

## 3. Results

### 3.1. Cohort Characteristics

The 3416 statin-naive patients had a mean age of 69.0 ± 12.7 years, and 1999 (58.5%) were male. Detailed demographic, comorbidity, and lipid panel characteristics are summarized in Table 1.

For the primary outcome (severe stenosis), more severe atherosclerosis was associated with older age, male sex, and a higher prevalence of hypertension, diabetes, prior stroke, and prior ischemic heart disease (Table 2). Demographic and lipid distributions for the burden-based secondary outcomes (carotid plaque score and number of stenotic intracranial vessels) showed similar patterns and are presented in Supplementary Tables S2 and S3. ApoA1 decreased across the normal, mild, and severe groups (median 1.36, 1.26, and 1.23 g/L, respectively; *p* < 0.0001), whereas ApoB differed little across groups. Accordingly, the ApoB/A1 ratio increased across severity groups (median 0.70, 0.75, and 0.79, respectively; *p* < 0.0001), largely driven by lower ApoA1 levels. The same monotonic patterns for ApoA1 and the ApoB/A1 ratio were observed for both burden-based secondary outcomes (Tables S2 and S3). Total cholesterol and LDL-C showed no clear positive gradient across increasing atherosclerosis severity.

### 3.2. Independent Associations of Lipid Biomarkers with Atherosclerosis Severity

In multivariable ordinal logistic regression, the ApoB/A1 ratio was strongly and independently associated with the primary outcome of severe stenosis (per 1-SD aOR 1.24, 95% CI 1.15–1.35; *p* < 0.0001). The protective association of ApoA1 was also pronounced for this outcome (aOR 0.86; *p* = 0.0002), whereas ApoB and LDL-C showed weaker associations (Table 3). Total cholesterol, triglycerides, and HDL-C were not consistently associated with severity after adjustment.

### 3.3. Incremental Discrimination of the ApoB/A1 Ratio

**Figure 2:**
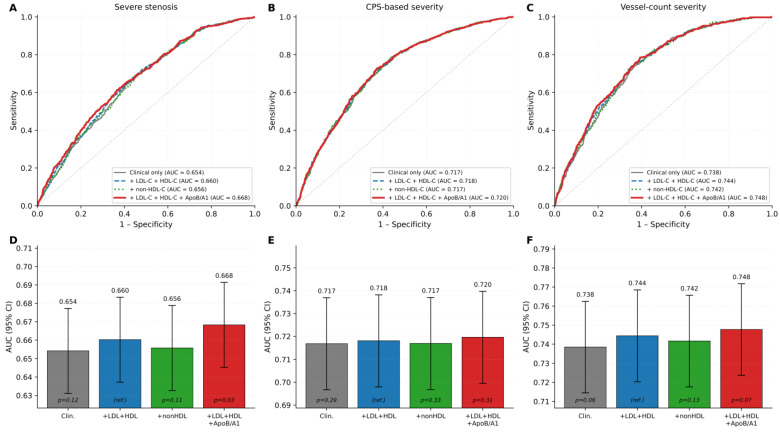
Receiver-operating-characteristic curves comparing discrimination across four models: clinical covariates only, clinical + LDL-C + HDL-C (conventional lipid panel), clinical + non-HDL-C (alternative lipid panel), and clinical + LDL-C + HDL-C + ApoB/A1 ratio (fully augmented model corresponding to the comparator in Table 4). The clinical + non-HDL-C curve (green dotted line) is shown for comparison and corresponds to the alternative reference model in Supplementary Table S5. The fully augmented model (red curve) corresponds to the incremental-discrimination comparison reported in Table 4. Panels show (**A**) the primary outcome (severe stenosis ≥ 50%) and (**B**,**C**) the burden-based secondary outcomes (CPS-based severity and vessel-count severity). The diagonal gray dashed line represents the line of no discrimination (reference line, AUC = 0.5). Panels (**D**–**F**) show the corresponding AUC values with 95% confidence intervals (DeLong variance) for the same four models; the *p*-value annotated for each non-reference bar is from the DeLong test against the LDL-C + HDL-C reference model. The AUC bar charts in Panels (**D**–**F**) make the numeric differences and their statistical uncertainty explicit. Outcomes: (**A**,**D**) primary outcome (severe stenosis ≥ 50%), (**B**,**E**) CPS-based severity, (**C**,**F**) vessel-count severity.

Adding the ApoB/A1 ratio to a reference model containing clinical covariates plus LDL-C and HDL-C (yielding the fully augmented model in Figure 2) significantly improved discrimination of severe stenosis on every metric examined: model fit improved (likelihood ratio χ^2^ = 12.59, *p* = 0.0004); risk reclassification improved (continuous NRI +0.142, 95% CI 0.053–0.227; IDI +0.0058, 0.0026–0.0088); and AUC increased from 0.660 to 0.668 (ΔAUC +0.008; DeLong *p* = 0.026; Table 4).

Findings for the burden-based secondary outcomes were directionally consistent with the primary analysis. The ApoB/A1 ratio was significantly associated with the CPS-based severity (aOR 1.15 per 1-SD, 95% CI 1.07–1.24; *p* = 0.0001) and the vessel-count severity (aOR 1.17, 1.08–1.27; *p* = 0.0001) (Table 3). Adding the ApoB/A1 ratio to the conventional lipid model significantly improved model fit (LRT *p* = 0.015 for CPS and *p* = 0.019 for vessel count) and yielded statistically significant NRI and IDI for both outcomes (Table 4); the AUC improvements were small and did not reach statistical significance for either secondary outcome (Figure 2).

### 3.4. Non-Linearity, Subgroup, Sensitivity, and ApoA1-Alone Analyses

Restricted cubic spline analyses showed no significant non-linearity between the ApoB/A1 ratio and the outcomes (Supplementary Figure S1). The association of ApoB/A1 with the primary outcome was consistent across prespecified subgroups, with no significant interactions (Supplementary Figure S2). The results were also robust in sensitivity analyses restricted to patients with both CUS and cerebrovascular imaging and using tertile-based severity cutoffs, as well as across alternative reference lipid models (Supplementary Tables S4 and S5).

To examine whether the observed signal was primarily driven by ApoA1 alone, we performed additional sensitivity analyses using the same reference model. For the primary outcome, adding ApoA1 alone improved model fit and reclassification but did not significantly improve AUC (0.660 to 0.664; DeLong *p* = 0.330). Adding the ApoB/A1 ratio produced the largest AUC increment (0.660 to 0.668; DeLong *p* = 0.026), with significant improvement in reclassification and model fit. However, the ApoA1-extended and ApoB/A1-extended models were not statistically distinguishable in direct comparison (DeLong *p* = 0.282). Adding ApoB to an ApoA1-containing model further improved model fit and reclassification, although the AUC increment remained small. Full results are provided in Supplementary Table S6. Findings for the burden-based secondary outcomes were directionally consistent.

## 4. Discussion

In this analysis of 3416 high-risk statin-naive patients with concurrently assessed extracranial and intracranial arteries, the ApoB/A1 ratio was independently associated with stenosis severity and modestly improved discrimination of severe stenosis beyond a conventional lipid panel based on LDL-C and HDL-C. The improvement was statistically significant on every contemporary measure of incremental discrimination: model fit (likelihood ratio test), reclassification (NRI and IDI), and AUC (DeLong test). Findings for the burden-based secondary outcomes (CPS and number of stenotic intracranial vessels) were directionally consistent, with significant NRI, IDI, and LRT but non-significant AUC improvement. The convergence of evidence across complementary outcome definitions supports the robustness of the primary finding.

Our findings extend prior work showing that the ApoB/A1 ratio reflects intracranial arterial stenosis [13,14] and carotid atherosclerosis [11,12]. Prior studies have often evaluated carotid atherosclerosis using intima-media thickness or binary plaque presence [12,21], whereas intracranial atherosclerosis has commonly been defined by the presence of significant stenosis, often using a ≥50% threshold [22]. By contrast, our analysis incorporated a primary stenosis-severity outcome together with burden-based secondary outcomes, including CPS and the number of stenotic intracranial vessels. This design allowed us to examine whether the ApoB/A1 ratio was associated not only with clinically significant stenosis but also with broader atherosclerotic burden. Several additional features strengthen the present study. First, the relatively large sample size and restriction to statin-naive patients minimized pharmacologic distortion of lipid metabolism and improved comparability of lipid biomarkers. Second, the simultaneous assessment of extracranial and intracranial arteries allowed us to examine whether the association of ApoB/A1 was consistent across two clinically relevant vascular beds. Finally, the use of incremental-discrimination metrics and sensitivity analyses allowed us to evaluate whether ApoB/A1 added information beyond conventional lipid markers rather than relying solely on odds-ratio comparisons.

The absolute AUC improvement after adding the ApoB/A1 ratio was modest (+0.008 for the primary outcome), likely because the reference model already included strong clinical predictors of atherosclerosis severity. Nevertheless, the consistent improvement in reclassification, model fit, and alternative reference models suggests that the ApoB/A1 ratio provided information not fully captured by conventional lipid markers. Thus, the clinical implication should be interpreted as incremental rather than transformative, with potential utility in refining risk stratification among high-risk patients undergoing carotid or cerebrovascular evaluation.

It is important to emphasize the scope of inference of the present study. Approximately 80% of the cohort had a documented history of stroke, and all patients were undergoing clinically indicated carotid or cerebrovascular evaluation; the cohort is therefore representative of a high-risk, hospital-based clinical population rather than of the general population. Our results support the interpretation that, within such a population, the ApoB/A1 ratio provides incremental discrimination of severe atherosclerosis beyond a conventional lipid panel. They do not, however, support inference about the value of the ApoB/A1 ratio as a screening tool in asymptomatic individuals or its normative variation in healthy individuals; both questions require prospective, population-based studies.

The attenuated LDL-C gradient across severity strata should be interpreted in light of the clinical composition of the reference group. In this study, “normal” denoted no definite stenosis on clinically indicated vascular imaging, not a healthy control state. Many patients in this group had vascular risk factors or prior cerebrovascular disease and underwent lipid profiling and vascular assessment for clinical reasons. Therefore, LDL-C levels in the reference group may already have reflected a high-risk clinical background, reducing the apparent contrast in LDL-C between groups. This does not negate the established role of LDL-C in atherosclerosis; rather, it suggests that in a high-risk, statin-naive hospital-based cohort, apolipoprotein-based markers, particularly the balance between ApoB and ApoA1, may capture residual variation in atherosclerotic burden not fully reflected by LDL-C alone.

The lipid distributions across severity strata provide additional insight into why the ApoB/A1 ratio remained informative. ApoB showed little difference across severity groups in unadjusted comparisons, whereas ApoA1 declined monotonically with increasing severity. However, the association between ApoB and stenosis severity became evident after adjustment for age and vascular risk factors, suggesting that the crude ApoB distribution was influenced by the high-risk clinical profile of the cohort. Consequently, the elevation of the ApoB/A1 ratio in severe disease appeared to be driven mainly by lower ApoA1 levels with additional adjusted contribution from ApoB. This finding suggests that the ApoB/A1 ratio may capture the balance between atherogenic and protective lipoprotein pathways more effectively than either component alone. ApoA1 alone captured a substantial part of the signal, particularly for model fit and reclassification. Therefore, our findings should not be interpreted as proving the clear superiority of the ApoB/A1 ratio over ApoA1 alone. Nevertheless, the ratio-based model produced the largest AUC increment over the conventional lipid model and was the only extension that achieved a DeLong-significant AUC improvement for the primary outcome. In addition, adding ApoB to an ApoA1-containing model provided modest improvement in model fit and reclassification. These findings support retaining the ApoB/A1 ratio as an integrated marker of atherogenic and anti-atherogenic balance, while emphasizing that its incremental clinical value is modest.

Carotid and intracranial arteries have distinct anatomical and hemodynamic characteristics. The carotid artery has a thicker vessel wall and is exposed to turbulent flow at the bifurcation, which may promote LDL-C deposition and plaque formation [23]. In contrast, intracranial arteries have thinner walls with fewer smooth-muscle cells and elastic fibers and may be particularly vulnerable to hypertension-mediated endothelial injury [24]. Despite these differences, both vascular beds share key pathogenic processes, including endothelial injury, LDL-C infiltration, oxidation, and inflammatory activation [25]. The consistent association of ApoB/A1 with atherosclerosis severity across both vascular beds is biologically plausible in this shared pathophysiologic context.

Several limitations should be considered. First, the cross-sectional, observational design precludes causal inference. Longitudinal studies are needed to establish whether changes in ApoB/A1 predict atherosclerosis progression. Second, this is a single-center cohort of one ethnic population, limiting generalizability. Multi-center studies using a common data model would strengthen external validity. Third, our cohort by design lacked a healthy control group, because carotid duplex ultrasonography and cerebrovascular imaging are not performed on asymptomatic individuals as a matter of routine practice and would be difficult to justify ethically in a healthy research volunteer. Approximately 80% of patients had a history of stroke, reflecting the hospital-based nature of the cohort. Our results should therefore be interpreted as describing the discriminative value of the ApoB/A1 ratio within a high-risk hospital-based cohort, and not as characterizing normative variation of the ApoB/A1 ratio in the general population. Fourth, although potential confounders were adjusted, smoking and alcohol use were ascertained from clinical records and may be subject to underreporting. Adiposity was captured only through BMI; waist circumference and waist-to-hip ratio, which directly reflect central adiposity and may better capture adiposity-related cardiovascular risk, were not consistently available in the underlying electronic health records. Dietary habits and genetic factors were also unavailable. Residual confounding by unmeasured adiposity, diet, or genetic background, therefore, cannot be excluded. Fifth, atherosclerosis severity was determined from rule-based text parsing of clinical imaging reports rather than independent re-measurement or blinded re-adjudication. Although the extraction rules were refined through manual review during data cleaning, formal validation with agreement statistics was not performed. Therefore, our analyses reflect severity as captured in clinical practice rather than centrally re-graded measurements. Future studies should include blinded centralized image review with quantitative stenosis grading. Sixth, no universally accepted severity cutoffs exist for the carotid plaque score or the number of stenotic intracranial vessels. We used data-driven median-based cutoffs and confirmed robustness with tertile-based sensitivity analyses but acknowledge that alternative classifications could yield somewhat different effect estimates for the secondary outcomes.

## 5. Conclusions

In this high-risk, hospital-based cohort of statin-naive patients undergoing carotid and/or cerebrovascular evaluation, the ApoB/A1 ratio was independently associated with atherosclerosis severity and provided modest incremental discrimination of severe stenosis beyond a conventional lipid panel. Given the small absolute improvement in discrimination, our findings support the ApoB/A1 ratio as an incremental refinement of risk stratification within similar high-risk clinical populations, rather than as a general-population screening tool or as a routine replacement for conventional lipid measurement. Prospective and, where possible, multicenter external validation is required before any change in clinical practice can be recommended.

## Figures and Tables

**Table 1:** Demographic and clinical characteristics of the study cohort.

Variable	Total (N = 3416)
Sex	
Female, n (%)	1417 (41.5)
Male, n (%)	1999 (58.5)
Age, years	69.0 ± 12.7
BMI, kg/m^2^	24.04 ± 3.43
Hypertension, n (%)	2158 (63.2)
Diabetes mellitus, n (%)	1014 (29.7)
Chronic kidney disease, n (%)	75 (2.2)
Stroke history, n (%)	2741 (80.2)
Ischemic heart disease, n (%)	201 (5.9)
Alcohol consumption, n (%)	992 (29.0)
Current smoker, n (%)	718 (21.0)
Apolipoprotein A1, g/L *	1.27 (1.11–1.44)
Apolipoprotein B, g/L *	0.95 (0.77–1.14)
Total cholesterol, mmol/L *	4.55 (3.88–5.28)
HDL-C, mmol/L *	1.14 (0.96–1.37)
LDL-C, mmol/L *	2.77 (2.17–3.36)
Triglyceride, mmol/L *	1.20 (0.86–1.66)
Non-HDL-C, mmol/L *	3.36 (2.72–4.06)
Apolipoprotein B/A1 ratio *	0.75 (0.58–0.94)
Carotid plaque score (n = 2794) *	2.30 (0.00–3.60)
No. stenotic vessels (n = 2422) *	1 (0–3)

Continuous variables are presented as mean ± standard deviation. * Median (interquartile range). BMI, body mass index; HDL-C, high-density lipoprotein cholesterol; LDL-C, low-density lipoprotein cholesterol.

**Table 2:** Demographic, clinical, and lipid characteristics according to stenosis severity in patients with cerebrovascular imaging.

Variable	Normal (n = 837)	Mild (n = 888)	Severe (n = 697)	*p*-Value
Sex, male, n (%)	442 (52.8)	534 (60.1)	442 (63.4)	<0.0001
Age, years	64.7 ± 12.4	71.5 ± 11.9	70.9 ± 12.7	<0.0001
BMI, kg/m^2^	24.2 ± 3.5	24.2 ± 3.4	23.8 ± 3.4	0.088
Hypertension, n (%)	389 (46.5)	596 (67.1)	492 (70.6)	<0.0001
Diabetes, n (%)	162 (19.4)	290 (32.7)	236 (33.9)	<0.0001
CKD, n (%)	6 (0.7)	20 (2.3)	20 (2.9)	0.006
Stroke history, n (%)	489 (58.4)	719 (81.0)	621 (89.1)	<0.0001
IHD history, n (%)	13 (1.6)	49 (5.5)	53 (7.6)	<0.0001
Alcohol, n (%)	242 (28.9)	245 (27.6)	226 (32.4)	0.102
Smoking, n (%)	166 (19.8)	182 (20.5)	166 (23.8)	0.132
Apolipoprotein A1, g/L	1.36 (1.18–1.53)	1.26 (1.11–1.43)	1.23 (1.09–1.40)	<0.0001
Apolipoprotein B, g/L	0.94 (0.79–1.12)	0.95 (0.78–1.15)	0.95 (0.79–1.18)	0.289
Total cholesterol, mmol/L	4.71 (3.98–5.40)	4.53 (3.90–5.23)	4.55 (3.80–5.33)	0.013
HDL-C, mmol/L	1.22 (1.01–1.45)	1.12 (0.96–1.34)	1.11 (0.93–1.32)	<0.0001
LDL-C, mmol/L	2.82 (2.22–3.36)	2.73 (2.22–3.28)	2.79 (2.17–3.44)	0.37
Triglyceride, mmol/L	1.20 (0.85–1.68)	1.21 (0.88–1.72)	1.22 (0.88–1.63)	0.598
Non-HDL-C, mmol/L	3.41 (2.79–4.11)	3.36 (2.74–4.01)	3.36 (2.69–4.14)	0.526
Apolipoprotein B/A1 ratio	0.70 (0.56–0.88)	0.75 (0.59–0.95)	0.79 (0.62–1.00)	<0.0001

Values are presented for 2422 patients with available cerebrovascular imaging. Stenosis severity was categorized as normal, mild (<50% stenosis), or severe (≥50% stenosis, including occlusion). Continuous variables are presented as mean ± SD or median (IQR); categorical variables as n (%). *p*-values were calculated using analysis of variance (normally distributed continuous variables), Kruskal–Wallis test (skewed continuous variables), or chi-squared test (categorical variables). BMI, body mass index; CKD, chronic kidney disease; IHD, ischemic heart disease; HDL-C, high-density lipoprotein cholesterol; LDL-C, low-density lipoprotein cholesterol.

**Table 3:** Adjusted odds ratios per 1-SD increase in lipid biomarkers for atherosclerosis severity.

Biomarker	Primary: Severe Stenosis aOR (95% CI)	*p*	Secondary: CPS-Based aOR (95% CI)	*p*	Secondary: Vessel-Count aOR (95% CI)	*p*
ApoA1	0.86 (0.79–0.93)	0.0002	0.93 (0.86–1.00)	0.053	0.94 (0.86–1.02)	0.141
ApoB	1.18 (1.09–1.28)	<0.0001	1.10 (1.02–1.19)	0.01	1.18 (1.08–1.28)	<0.0001
Total cholesterol	1.06 (0.98–1.15)	0.135	1.04 (0.96–1.12)	0.351	1.12 (1.03–1.21)	0.008
HDL-C	0.90 (0.83–0.98)	0.013	0.93 (0.86–1.00)	0.044	0.96 (0.88–1.04)	0.33
LDL-C	1.13 (1.04–1.22)	0.002	1.07 (1.00–1.16)	0.061	1.13 (1.04–1.23)	0.003
Triglyceride	1.01 (0.93–1.10)	0.845	1.06 (0.98–1.14)	0.18	1.06 (0.97–1.15)	0.221
Non-HDL-C	1.10 (1.01–1.19)	0.021	1.06 (0.98–1.14)	0.123	1.14 (1.05–1.23)	0.002
ApoB/A1 ratio	1.24 (1.15–1.35)	<0.0001	1.15 (1.07–1.24)	0.0001	1.17 (1.08–1.27)	0.0001

Each lipid biomarker was entered into a separate ordinal logistic regression model, adjusted for age, sex, BMI, hypertension, diabetes, chronic kidney disease, prior stroke, prior ischemic heart disease, alcohol consumption, and smoking. aOR, adjusted odds ratio per 1-standard-deviation increase; CI, confidence interval; CPS, carotid plaque score.

**Table 4:** Incremental discrimination of ApoB/A1 ratio over a conventional lipid panel.

Outcome	AUC (LDL + HDL)	AUC (+ApoB/A1)	ΔAUC	DeLong *p*	NRI (95% CI)	IDI (95% CI)	LRT *p*
Primary: severe stenosis	0.660	0.668	+0.008	0.026	+0.142 (0.053–0.227)	+0.0058 (0.0026–0.0088)	0.0004
Secondary: CPS ≥ median	0.718	0.720	+0.002	0.311	+0.112 (0.032–0.196)	+0.0022 (0.0004–0.0040)	0.015
Secondary: vessel ≥ median	0.744	0.748	+0.003	0.072	+0.203 (0.104–0.303)	+0.0025 (0.0004–0.0046)	0.019

Reference model: clinical covariates + LDL-C + HDL-C. Comparator model adds ApoB/A1 ratio. NRI, continuous net reclassification improvement; IDI, integrated discrimination improvement; LRT, likelihood ratio test.

## Data Availability

The datasets analyzed during the current study are not publicly available because they contain protected health information governed by the data-use agreement of the National Health Insurance Service Ilsan Hospital clinical data warehouse, but de-identified data are available from the corresponding author on reasonable request and subject to institutional approval.

## Supplementary Materials

The following supporting information can be downloaded at: https://www.mdpi.com/article/10.3390/jcm15145598/s1, Supplementary Methods: Rule-based extraction of stenosis severity and intracranial vessel count from imaging reports; Table S1: Representative report expressions and rule-based assignments; Table S2: Comparison of demographic, clinical, and lipid variables across carotid plaque score categories; Table S3: Comparison of demographic, clinical, and lipid variables across number of stenotic intracranial vessels; Table S4: Sensitivity analyses for the association between Apolipoprotein B/A1 ratio and atherosclerosis severity; Table S5: Incremental discrimination of the Apolipoprotein B/A1 ratio across alternative base-model specifications; Table S6: Additional analyses comparing ApoA1, ApoB, and the ApoB/A1 ratio as extensions of the reference model; Figure S1: Restricted cubic spline plots of the Apolipoprotein B/A1 ratio and atherosclerosis severity; Figure S2: Forest plot of subgroup-specific associations between the Apolipoprotein B/A1 ratio and atherosclerosis severity.

## Abbreviations

ApoA1, apolipoprotein A1; ApoB, apolipoprotein B; aOR, adjusted odds ratio; AUC, area under the receiver operating characteristic curve; BMI, body mass index; CI, confidence interval; CKD, chronic kidney disease; CPS, carotid plaque score; CTA, computed tomography angiography; CUS, carotid duplex ultrasonography; HDL-C, high-density lipoprotein cholesterol; IDI, integrated discrimination improvement; IHD, ischemic heart disease; IQR, interquartile range; LDL-C, low-density lipoprotein cholesterol; LRT, likelihood ratio test; MRA, magnetic resonance angiography; NRI, net reclassification improvement; ROC, receiver operating characteristic; SD, standard deviation.
