# Supplementary material for: Apolipoprotein B/A1 Ratio Improves Discrimination of Severe Atherosclerosis Beyond Conventional Lipid Markers in High-Risk Statin-Naive Patients
Source: J Clin Med. 2026 Jul 16;15(14):5598. doi: 10.3390/jcm15145598 (PMC13412671; doi:10.3390/jcm15145598)
Supplement: Supplementary file 1 [file jcm-15-05598-s001.zip › jcm-4414931-supplementary.pdf]

Supplementary Material

Supplementary Methods

Rule-based extraction of stenosis severity and intracranial vessel count from imaging reports

Vascular stenosis information was extracted from clinical MRA or CTA reports using a rule-based text-parsing approach. The purpose of the extraction was to convert qualitative radiology report descriptions into structured indicators of stenosis severity and the number of stenotic intracranial vessels.

1. Initial identification of reports without definite stenosis

Reports were first screened for standardized negative expressions indicating no definite stenosis or occlusion. Representative expressions included "No significant stenosis," "No significant stenosis or occlusion," "No definite stenosis or occlusion on major vessels," "No significant stenosis, occlusive lesion or aneurysm in intra/extracranial major arteries," "Negative MRA study," and "MR angiography is grossly normal." Reports containing these expressions without any additional definite stenosis descriptor were classified as having no definite stenosis. After removal of these negative expressions, reports were retained for further review if they contained stenosis-related terms, including stenosis, stenoses, occlusion, atherosclerotic, or atherosclerosis. Common typographical variants were also considered during rule refinement.

2. Text preprocessing and exclusion of non-stenosis vascular expressions

Before vessel counting, the report text was standardized by harmonizing common abbreviations and segment names. The term "both" was treated as equivalent to "bilateral." Segment-level expressions were mapped to their parent vessels: M1, M2, and M3 were mapped to MCA; A1, A2, and A3 to ACA; P1, P2, and P3 to PCA; V4 to distal vertebral artery; and cavernous, paraclinoid, cavernous, and distal ICA to distal internal carotid artery. Basilar artery and vertebrobasilar expressions were mapped to BA when the basilar segment was described.

Expressions referring to infarct territory or parenchymal lesion location were excluded before vessel counting to avoid misclassifying lesion location as arterial stenosis. These included expressions such as MCA territory, ACA territory, PCA territory, MCA region, ACA region, PCA region, and similar territory-based or region-based descriptions. Extracranial terms, including CCA, carotid bulb, carotid bifurcation, proximal ICA, proximal cervical ICA, proximal VA, VA os, and VA orifice, were not counted as intracranial stenotic vessels. However, when these expressions appeared in a sentence that also contained eligible intracranial vessel terms, the intracranial vessel terms were still evaluated.

3. Mapping of intracranial vessels

Intracranial vessels were mapped into 11 analyzable vessels: right and left distal internal carotid arteries, right and left middle cerebral arteries, right and left anterior cerebral arteries, right and left posterior cerebral arteries, right and left distal vertebral arteries, and the basilar artery. The basilar artery was counted as one unpaired vessel.

The following mappings were applied:

- Distal internal carotid artery: distal ICA, cavernous ICA, paraclinoid ICA, cavernous ICA, intracranial ICA.

- Middle cerebral artery: MCA, M1, M2, M3.
- Anterior cerebral artery: ACA, A1, A2, A3.
- Posterior cerebral artery: PCA, P1, P2, P3.
- Distal vertebral artery: distal VA, intracranial VA, V4.
- Basilar artery: BA, basilar artery, vertebrobasilar artery when the basilar artery was described.

4. Laterality and bilateral vessel-counting rules

For paired vessels, laterality was assigned using right, left, Rt, Lt, or bilateral expressions. Bilateral expressions were counted as involvement of both right and left vessels. For example, “bilateral MCAs” was counted as two stenotic vessels, and “bilateral ICAs, MCAs, and PCAs” was counted as six stenotic vessels. If bilateral involvement of a parent vessel had already been counted, subsequent right- or left-sided mentions of the same parent vessel in the same report were not counted again.

For unilateral expressions, a parent vessel was counted once per side. For example, “right M1 stenosis” and “right MCA stenosis” were both mapped to right MCA and counted once. Similarly, multiple mentions of M1, M2, or MCA on the same side were collapsed into a single right or left MCA count. The same collapsing rule was applied to ACA, PCA, distal vertebral artery, and distal ICA segment-level expressions.

5. Stenosis severity assignment

Stenosis severity was assigned using the highest severity descriptor identified in the report. Reports with no definite stenosis after application of the negative-expression rules were classified as degree 0. Among reports with definite stenosis, degree 1 was assigned when stenosis was described as mild, mild-to-moderate, luminal irregularity, atherosclerotic irregularity, or stenosis without a severe, significant, or occlusive descriptor. Degree 2 was assigned when the report contained severe stenosis, significant stenosis, high-grade stenosis, near occlusion, occlusion, total occlusion, or steno-occlusion. When multiple severity descriptors were present, the highest severity category was retained.

6. Derivation of the vessel-count variable

For each patient, the vessel-count variable was calculated as the sum of the involved intracranial vessels after collapsing segment-level terms into parent vessels and removing duplicate mentions. The maximum possible count was 11: five paired intracranial vessel groups on each side plus the basilar artery. Extracranial carotid or vertebral lesions were not included in the intracranial vessel count.

7. Rule refinement

The rule set was developed through iterative manual review of imaging reports. During data cleaning, report expressions that produced discordant or ambiguous extraction results were reviewed, and the rules were refined accordingly. The manually reviewed reports were used for rule development and refinement rather than as an independent validation dataset; therefore, formal agreement statistics such as kappa coefficients were not calculated.

Supplementary Table S1. Representative report expressions and rule-based assignments

Expression from imaging reports	Rule-based assignment	Rationale
Normal brain MRI and MRA.	Degree 0; vessel count 0	Standard normal angiographic expression without definite stenosis
MR angiography is grossly normal.	Degree 0; vessel count 0	Negative MRA expression indicating no definite vascular stenosis
Negative MRA study.	Degree 0; vessel count 0	Standard negative angiographic expression
Stenosis in the proximal M1 segment of the left MCA, mild.	Degree 1; left MCA counted once	M1 was mapped to MCA; mild stenosis was classified as non-severe
Mild to moderate stenosis right distal ICA.	Degree 1; right distal ICA counted once	Distal ICA was treated as an intracranial ICA segment; mild-to-moderate stenosis was classified as non-severe
Focal stenoses at the proximal M2 segments of the right MCA, significant.	Degree 2; right MCA counted once	M2 was mapped to MCA; significant stenosis was classified as severe stenosis
No change of focal severe stenosis at left M2, A2, and P2.	Degree 2; left MCA, left ACA, and left PCA counted	M2, A2, and P2 were mapped to MCA, ACA, and PCA, respectively; severe stenosis was classified as degree 2
Occlusion of right distal VA.	Degree 2; right distal VA counted once	Occlusion was included in the severe stenosis/occlusion category
Occlusion of right distal M1 segment and left inferior M2 segment.	Degree 2; right MCA and left MCA counted	M1 and M2 were mapped to MCA; right and left MCA were counted separately
Multiple focal stenoses are seen in right M1, right A2, right P1, proximal M2 of left MCA inferior division, left A1 and distal left vertebral artery.	Right MCA, right ACA, right PCA, left MCA, left ACA, and left distal VA counted	Segment-level terms were mapped to parent vessels and counted once per side
Atherosclerosis in the bilateral ICAs, MCAs, ACAs, PCAs…	Bilateral ICAs, MCAs, ACAs, and PCAs counted	Bilateral paired-vessel expressions were counted as right and left involvement for each parent vessel
Atherosclerosis on bilateral ICAs BA.	Bilateral distal ICAs and BA counted	Bilateral ICAs were counted as two paired vessels; BA was counted as one unpaired vessel

Luminal irregularities from atherosclerosis are noted in the bilateral cavernous ICAs, MCAs, ACAs, PCAs, and left proximal cervical ICA…	Bilateral distal ICAs, MCAs, ACAs, and PCAs counted; proximal cervical ICA not counted as intracranial vessel	Cavernous ICAs were mapped to distal ICAs; proximal cervical ICA was treated as extracranial and excluded from intracranial vessel count
Stenoses are also noted at the junction of right petrous-cavernous ICA, proximal basilar artery, bilateral V4 segments…	Right distal ICA, BA, and bilateral distal VAs counted	Petrous-cavernous ICA was mapped to distal ICA; proximal basilar artery to BA; V4 to distal VA
Recent infarction with partial hemorrhagic transformation is noted in the left MCA region.	Not counted as MCA stenosis	Region expression was interpreted as parenchymal lesion location, not arterial stenosis
Acute infarctions in right MCA territory.	Not counted as MCA stenosis	Territory expression was excluded before vessel counting
Fetal origin of right PCA.	Not counted as PCA stenosis	Developmental variant without definite stenosis descriptor
Hypoplastic right VA.	Not counted as vertebral artery stenosis unless accompanied by a definite stenosis or occlusion descriptor	Anatomic variant alone was not considered stenosis
A small aneurysm is noted at the right paraclinoid ICA.	Not counted as distal ICA stenosis	Aneurysm without stenosis descriptor was not classified as stenosis
Severe stenosis in the distal M1 segment of the left MCA. Segmental stenosis is also noted in the right proximal ICA at the carotid bulb level.	Left MCA counted; carotid bulb lesion not counted for intracranial vessel count	Intracranial MCA stenosis was retained; extracranial carotid bulb/proximal ICA lesion was excluded from intracranial vessel count

Representative report expressions and their assigned severity and intracranial vessel-count categories following the rule-based extraction described in the Supplementary Methods. The excerpts have been shortened to relevant phrases for clarity; the full de-identified reports were parsed according to the rule set.

Supplementary Table S2. Comparison of demographic, clinical, and lipid variables across carotid plaque score categories

Variable	Normal (n=1,109)	Mild (n=859)	Severe (n=826)	p-value
Sex, male, n (%)	617 (55.6)	520 (60.5)	586 (70.9)	<.0001
Age, years	63.4 ± 13.0	71.4 ± 11.9	73.7 ± 10.6	<.0001
BMI, kg/m²	24.4 ± 3.5	23.9 ± 3.4	23.7 ± 3.4	<.0001
Hypertension, n (%)	684 (61.7)	635 (73.9)	608 (73.6)	<.0001
Diabetes, n (%)	270 (24.3)	303 (35.3)	328 (39.7)	<.0001
CKD, n (%)	15 (1.4)	27 (3.1)	25 (3.0)	0.014
Stroke history, n (%)	1,001 (90.3)	794 (92.4)	770 (93.2)	0.046
IHD history, n (%)	44 (4.0)	51 (5.9)	89 (10.8)	<.0001
Alcohol, n (%)	367 (33.1)	273 (31.8)	279 (33.8)	0.672
Smoking, n (%)	265 (23.9)	197 (22.9)	198 (24.0)	0.849
Apolipoprotein A1, g/L	1.27 (1.13–1.44)	1.26 (1.09–1.41)	1.21 (1.06–1.38)	<.0001
Apolipoprotein B, g/L	0.95 (0.78–1.13)	0.96 (0.78–1.17)	0.93 (0.75–1.12)	0.061
Total cholesterol, mmol/L	4.60 (3.90–5.28)	4.53 (3.80–5.30)	4.37 (3.75–5.07)	<.0001
HDL-C, mmol/L	1.14 (0.96–1.34)	1.14 (0.96–1.34)	1.09 (0.91–1.29)	<.0001
LDL-C, mmol/L	2.79 (2.20–3.36)	2.77 (2.17–3.39)	2.61 (2.07–3.23)	0.002
Triglyceride, mmol/L	1.22 (0.86–1.68)	1.21 (0.88–1.65)	1.18 (0.87–1.66)	0.516
Non-HDL-C, mmol/L	3.41 (2.79–4.06)	3.36 (2.66–4.11)	3.23 (2.59–3.93)	0.002
Apolipoprotein B/A1 ratio	0.74 (0.59–0.92)	0.77 (0.60–0.97)	0.77 (0.59–0.98)	0.044

Values are presented for 2,794 patients who underwent carotid duplex ultrasonography. Carotid plaque score severity was stratified using the median carotid plaque score among patients with any plaque (median = 3.0). Continuous variables are presented as mean ± SD or median (IQR); categorical variables as n (%). p-values from ANOVA, Kruskal–Wallis, or chi-squared test. BMI, body mass index; CKD, chronic kidney disease; IHD, ischemic heart disease.

Supplementary Table S3. Comparison of demographic, clinical, and lipid variables across number of stenotic intracranial vessels

Variable	Normal (n=1,132)	Mild (n=884)	Severe (n=406)	p-value
Sex, male, n (%)	626 (55.3)	553 (62.6)	239 (58.9)	0.005
Age, years	65.9 ± 12.6	70.6 ± 12.4	74.2 ± 11.0	<.0001
BMI, kg/m²	24.2 ± 3.5	24.0 ± 3.4	24.0 ± 3.3	0.308
Hypertension, n (%)	556 (49.1)	586 (66.3)	335 (82.5)	<.0001
Diabetes, n (%)	242 (21.4)	275 (31.1)	171 (42.1)	<.0001
CKD, n (%)	6 (0.5)	21 (2.4)	19 (4.7)	<.0001
Stroke history, n (%)	709 (62.6)	746 (84.4)	374 (92.1)	<.0001
IHD history, n (%)	23 (2.0)	52 (5.9)	40 (9.9)	<.0001
Alcohol, n (%)	324 (28.6)	280 (31.7)	109 (26.8)	0.149
Smoking, n (%)	217 (19.2)	228 (25.8)	69 (17.0)	<.001
Apolipoprotein A1, g/L	1.34 (1.15–1.51)	1.24 (1.10–1.41)	1.25 (1.09–1.42)	<.0001
Apolipoprotein B, g/L	0.95 (0.79–1.13)	0.93 (0.78–1.15)	0.99 (0.79–1.19)	0.182
Total cholesterol, mmol/L	4.65 (3.96–5.40)	4.47 (3.83–5.20)	4.60 (3.86–5.35)	0.012
HDL-C, mmol/L	1.19 (0.98–1.43)	1.11 (0.93–1.32)	1.12 (0.96–1.37)	<.0001
LDL-C, mmol/L	2.82 (2.25–3.39)	2.72 (2.20–3.28)	2.82 (2.18–3.44)	0.167
Triglyceride, mmol/L	1.20 (0.85–1.67)	1.22 (0.89–1.70)	1.23 (0.89–1.63)	0.454
Non-HDL-C, mmol/L	3.44 (2.77–4.09)	3.32 (2.74–4.03)	3.41 (2.72–4.16)	0.365
Apolipoprotein B/A1 ratio	0.71 (0.57–0.90)	0.77 (0.59–0.96)	0.81 (0.61–1.00)	<.0001

Values are presented for 2,422 patients with available cerebrovascular imaging. Vessel-count severity was stratified using the median number of stenotic intracranial vessels among patients with any stenosis (median = 3). Continuous variables are presented as mean ± SD or median (IQR); categorical variables as n (%). p-values from ANOVA, Kruskal–Wallis, or chi-squared test. BMI, body mass index; CKD, chronic kidney disease; IHD, ischemic heart disease.

Supplementary Table S4. Sensitivity analyses for the association between Apolipoprotein B/A1 ratio and atherosclerosis severity

Scenario	Outcome	N	aOR per 1-SD (95% CI)	p-value
Primary analysis	Severe stenosis	2,422	1.24 (1.15–1.35)	<.0001
Primary analysis	CPS-based severity	2,794	1.15 (1.07–1.24)	0.0001
Primary analysis	Vessel-count severity	2,422	1.17 (1.08–1.27)	0.0001
Both CUS+Cerebrovascular imaging available	Severe stenosis	1,800	1.25 (1.14–1.37)	<.0001
Both CUS+Cerebrovascular imaging available	CPS-based severity	1,800	1.12 (1.02–1.22)	0.015
Both CUS+Cerebrovascular imaging available	Vessel-count severity	1,800	1.15 (1.05–1.26)	0.002
Tertile-based cutoff	Severe stenosis	2,422	1.24 (1.15–1.35)	<.0001
Tertile-based cutoff	CPS-based severity	2,794	1.14 (1.06–1.23)	0.0003
Tertile-based cutoff	Vessel-count severity	2,422	1.17 (1.08–1.27)	0.0001

Values are adjusted odds ratios per 1-SD increase in the ApoB/A1 ratio across alternative analytic specifications. Models were adjusted for age, sex, BMI, hypertension, diabetes, chronic kidney disease, prior stroke, prior ischemic heart disease, alcohol consumption, and current smoking. CPS, carotid plaque score; CUS, carotid duplex ultrasonography.

Supplementary Table S5. Incremental discrimination of the Apolipoprotein B/A1 ratio across alternative base-model specifications

Reference model	AUC base	AUC ext	ΔAUC	DeLong p	NRI (95% CI)	IDI (95% CI)	LRT p
Main reference: Clinical + LDL-C + HDL-C	0.660	0.668	+0.008	0.026	+0.142 (0.053–0.227)	+0.0058 (0.0026–0.0088)	0.0004
Alternative A: Clinical + LDL-C + HDL-C + triglycerides	0.661	0.669	+0.008	0.025	+0.155 (0.069–0.242)	+0.0059 (0.0030–0.0093)	0.0003
Alternative B: Clinical + non-HDL-C	0.656	0.668	+0.013	0.011	+0.196 (0.105–0.286)	+0.0090 (0.0049–0.0130)	<0.0001

Analyses were performed for the primary outcome of severe stenosis (≥50%). Reference models comprise clinical covariates (age, sex, BMI, hypertension, diabetes, chronic kidney disease, prior stroke, prior ischemic heart disease, alcohol consumption, current smoking) plus the specified lipid variables. The comparator model in each row adds the ApoB/A1 ratio (1-SD–standardized). AUC base, AUC of the reference model; AUC ext, AUC of the reference model with the ApoB/A1 ratio added; ΔAUC = AUC ext − AUC base; NRI, continuous net reclassification improvement; IDI, integrated discrimination improvement; LRT, likelihood ratio test for the nested logistic model. The first row reproduces the main-analysis comparison (Clinical + LDL-C + HDL-C reference) as reported in Table 4 of the main text. Across all three reference panels, adding the Apolipoprotein B/A1 ratio significantly improved discrimination of the primary outcome on every metric examined.

Supplementary Table S6. Additional analyses comparing ApoA1, ApoB, and the ApoB/A1 ratio as extensions of the reference model

Base (reference) model: All analyses used the same reference model as main-text Table 4: clinical covariates plus LDL-C and HDL-C. Clinical covariates were age, sex, BMI, hypertension, diabetes, chronic kidney disease, prior stroke, prior ischemic heart disease, alcohol consumption, and current smoking. ApoA1, ApoB, and the ApoB/A1 ratio were standardized per 1 SD within each outcome-specific analytic cohort. Bootstrap 95% confidence intervals for NRI and IDI were estimated using 1,000 resamples, as in the main analysis.

Panel A. Extension of the reference model by ApoA1 alone, ApoB alone, or the ApoB/A1 ratio

Outcome	Extension	AUC (base)	AUC (ext)	ΔAUC	DeLong p	NRI (95% CI)	IDI (95% CI)	LRT p
Primary: severe stenosis (≥50%)	ApoA1	0.660	0.664	+0.004	0.330	+0.125 (0.035–0.214)	+0.0051 (0.0021–0.0083)	0.0002
	ApoB	0.660	0.662	+0.002	0.488	+0.067 (−0.025–0.159)	+0.0018 (0.0002–0.0033)	0.060
	ApoB/A1	0.660	0.668	+0.008	0.026	+0.142 (0.053–0.227)	+0.0058 (0.0026–0.0088)	0.0004
Secondary: CPS ≥ median	ApoA1	0.718	0.718	+0.000	0.860	+0.030 (−0.058–0.115)	+0.0003 (-0.0002–0.0007)	0.498
	ApoB	0.718	0.719	+0.001	0.391	+0.056 (−0.021–0.145)	+0.0007 (-0.0003–0.0017)	0.157
	ApoB/A1	0.718	0.720	+0.002	0.311	+0.112 (0.032–0.196)	+0.0022 (0.0004–0.0040)	0.015
Secondary: vessel ≥ median	ApoA1	0.744	0.745	+0.001	0.713	+0.057 (−0.045–0.160)	+0.0009 (-0.0005–0.0023)	0.176
	ApoB	0.744	0.747	+0.003	0.181	+0.167 (0.062–0.274)	+0.0020 (-0.0001–0.0042)	0.029
	ApoB/A1	0.744	0.748	+0.003	0.072	+0.203 (0.104–0.303)	+0.0025 (0.0004–0.0046)	0.019

AUC, area under the receiver operating characteristic curve; ΔAUC, change in AUC after adding the extension variable; DeLong p, DeLong-test p-value for AUC comparison against the reference model; NRI, continuous net reclassification improvement; IDI, integrated discrimination improvement; LRT, likelihood ratio test for the nested logistic model. n = 2,422 (primary and vessel-count); n = 2,794 (CPS).

Panel B. Head-to-head DeLong comparison between extended models

Outcome	Comparison (both extend base)	AUC (a)	AUC (b)	DeLong p
Primary: severe stenosis (≥50%)	base + ApoA1 vs. base + ApoB/A1	0.664	0.668	0.282
	base + ApoA1 vs. base + ApoB	0.664	0.662	0.624
	base + ApoB vs. base + ApoB/A1	0.662	0.668	0.016
Secondary: CPS ≥ median	base + ApoA1 vs. base + ApoB/A1	0.718	0.720	0.278
	base + ApoA1 vs. base + ApoB	0.718	0.719	0.492
	base + ApoB vs. base + ApoB/A1	0.719	0.720	0.530
Secondary: vessel ≥ median	base + ApoA1 vs. base + ApoB/A1	0.745	0.748	0.077
	base + ApoA1 vs. base + ApoB	0.745	0.747	0.381
	base + ApoB vs. base + ApoB/A1	0.747	0.748	0.625

Paired DeLong test comparing the ROC curves of two extended models sharing the same reference model. AUC (a) refers to the first-named model in each row.

Panel C. Nested analysis testing whether ApoB adds information beyond ApoA1

Outcome	AUC (base + ApoA1)	AUC (+ ApoB)	ΔAUC	DeLong p	NRI (95% CI)	IDI (95% CI)	LRT p
Primary: severe stenosis (≥50%)	0.664	0.667	+0.002	0.390	+0.095 (0.009–0.176)	+0.003 (0.001–0.005)	0.013
Secondary: CPS ≥ median	0.718	0.719	+0.001	0.346	+0.064 (−0.014–0.143)	+0.001 (0.000–0.002)	0.130
Secondary: vessel ≥ median	0.745	0.748	+0.003	0.136	+0.172 (0.070–0.279)	+0.003 (0.000–0.005)	0.017

Reference model here is clinical covariates + LDL-C + HDL-C + ApoA1. The extended model adds ApoB. All metrics as in panel (A).

Note. Panel A compares ApoA1 alone, ApoB alone, and the ApoB/A1 ratio as single-variable extensions of the reference model. Panel B provides head-to-head DeLong comparisons between extended models. Panel C evaluates whether ApoB adds information beyond a model that already contains ApoA1. Overall, the ApoB/A1 ratio showed the largest AUC increment for the primary outcome, but the ApoA1-extended and ApoB/A1-extended models were not statistically distinguishable in direct comparison; therefore, these findings support retaining the ratio as an integrated marker while indicating that its incremental value over ApoA1 alone is modest.

Supplementary Figure 1. Restricted cubic spline plots of the ApoB/A1 ratio and atherosclerosis severity

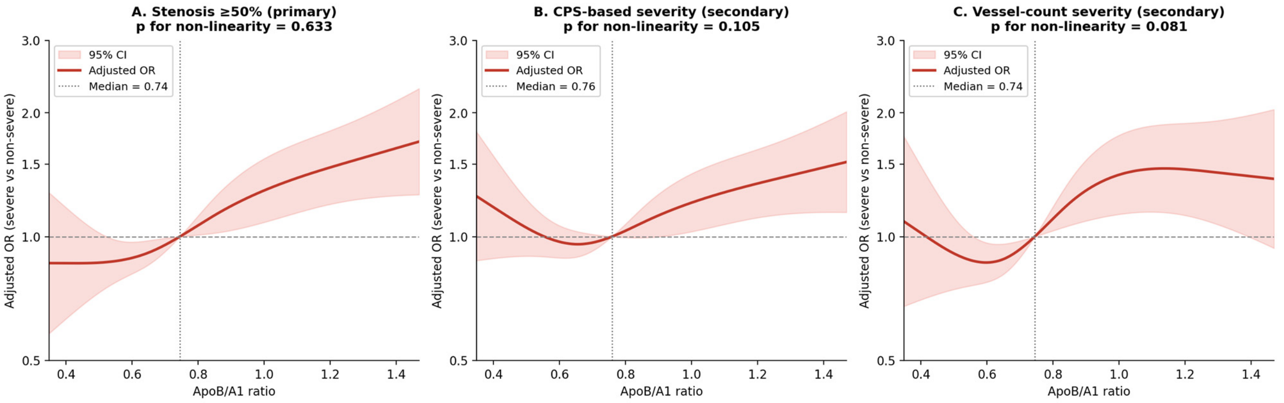

Adjusted for age, sex, BMI, hypertension, diabetes, CKD, prior stroke, prior IHD, alcohol, and smoking. Reference: cohort median ApoB/A1 ratio for each outcome cohort. Four knots at 5th/35th/65th/95th percentiles.

Supplementary Figure S1. Restricted cubic spline plots of the Apolipoprotein B/A1 ratio and atherosclerosis severity

Adjusted odds ratio for severe atherosclerosis (vs. non-severe) as a function of the Apolipoprotein B/A1 ratio, modeled with a restricted cubic spline using four knots at the 5th, 35th, 65th, and 95th percentiles, adjusted for age, sex, BMI, hypertension, diabetes, chronic kidney disease, prior stroke, prior ischemic heart disease, alcohol consumption, and current smoking. Panels show (A) the primary outcome of severe stenosis (≥50%), (B) CPS-based severity, and (C) vessel-count severity. The dotted vertical line marks the cohort median ApoB/A1 ratio. Non-linearity was not statistically significant for any outcome (p for non-linearity = 0.633, 0.105, and 0.081, respectively), supporting the use of linear terms in the primary ordinal models.

Supplementary Figure 2. Subgroup-specific associations between the ApoB/A1 ratio and atherosclerosis severity

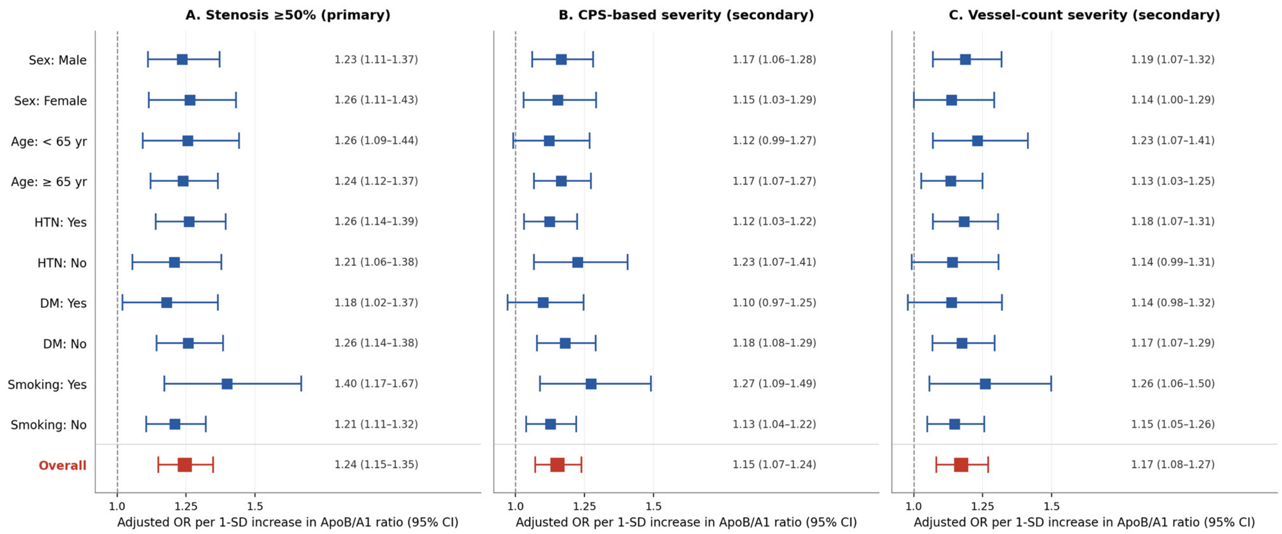

All interaction p-values > 0.05 across all subgroup × outcome combinations.

Supplementary Figure S2. Forest plot of subgroup-specific associations between the Apolipoprotein B/A1 ratio and atherosclerosis severity

Adjusted odds ratios per 1-standard-deviation increase in the Apolipoprotein B/A1 ratio, estimated within pre-specified subgroups of sex (male, female), age (<65, ≥65 years), hypertension (yes, no), diabetes mellitus (yes, no), and current smoking (yes, no). Panels show (A) the primary outcome of severe stenosis (≥50%), (B) CPS-based severity, and (C) vessel-count severity. Models were adjusted for the same covariates as the main analysis. The pooled overall estimate is shown in red. No subgroup×ApoB/A1 interaction was statistically significant for any outcome (all p interaction > 0.05).
